# Genome editing for inborn errors of metabolism: advancing towards the clinic

**DOI:** 10.1186/s12916-017-0798-4

**Published:** 2017-02-27

**Authors:** Jessica L. Schneller, Ciaran M. Lee, Gang Bao, Charles P. Venditti

**Affiliations:** 1 Department of Biomedical Engineering, SUNY Stony Brook, Stony Brook, NY USA; 2 Medical Genomics and Metabolic Genetics Branch, National Human Genome Research Institute, National Institutes of Health, Building 10, Room, 7N248A Bethesda, MD USA; 3 Department of Bioengineering, Rice University, 6500 Main Street, Houston, TX 77030 USA

**Keywords:** Inborn errors of metabolism, Genome editing, CRISPR/Cas9, Zinc-finger nucleases, Liver metabolic disorders

## Abstract

Inborn errors of metabolism (IEM) include many disorders for which current treatments aim to ameliorate disease manifestations, but are not curative. Advances in the field of genome editing have recently resulted in the in vivo correction of murine models of IEM. Site-specific endonucleases, such as zinc-finger nucleases and the CRISPR/Cas9 system, in combination with delivery vectors engineered to target disease tissue, have enabled correction of mutations in disease models of hemophilia B, hereditary tyrosinemia type I, ornithine transcarbamylase deficiency, and lysosomal storage disorders. These in vivo gene correction studies, as well as an overview of genome editing and future directions for the field, are reviewed and discussed herein.

## Background

Inborn errors of metabolism (IEM) are genetic disorders typically caused by an enzyme deficiency. As a consequence of the defect, insufficient conversion of substrate into metabolic product occurs, which can produce pathology by a variety of mechanisms, including the accumulation of toxic metabolites upstream of the block, reduction of essential downstream compounds, feedback inhibition or activation by the proximal metabolite on the same or different pathway, or abnormal alternative substrate metabolism [1]. Traditional therapies for IEM aim to reduce substrates, remove toxic intermediates, and/or supplement essential downstream products. Activation of alternative pathways for disposal of toxic intermediates is also employed, as in the case of urea cycle disorders [2], and for some conditions, enzyme replacement therapy is available. While an understanding of the pathophysiology of IEM has led to the development of more focused treatments, the correction of the underlying genetic mutation still remains as the ultimate therapeutic goal. With the advent of genome editing, a single treatment offering permanent and effective therapy may soon be realized. In this review, we discuss recent examples of in vivo genome editing for correction of preclinical models of IEM, disorders where the first clinical applications of genome editing may likely be implemented.

As the principal site for many intermediary metabolic reactions, the liver is the main target organ to correct for improving disease-related phenotypes [3]. Of the three major cell types in the liver, the majority of cells (~70%) are hepatocytes. The degree to which hepatocytes must be corrected to achieve therapeutic benefit for a given IEM depends on factors intrinsic to the perturbed biochemical pathway, and enzymopathy. Some disorders, such as hemophilia, require minimal activity to correct the associated bleeding propensity, while others, such as the proximal urea cycle disorder ornithine transcarbamylase (OTC) will require more activity, and larger numbers of cells corrected, to normalize metabolism [4]. Enzyme replacement via elective liver or combined liver-kidney transplantation is considered for conditions such as urea cycle disorders or organic acidemias when the clinical phenotype is severe [5–7]. Because most metabolic diseases are caused by loss-of-function mutations that occur in enzymes widely expressed in hepatocytes, gene therapy may thus represent a treatment option that could avoid the surgical complications of transplantation, yet provide the full benefits of liver replacement.

### Adeno-associated viruses (AAVs)

The ideal gene transfer vector will safely and efficiently deliver a therapeutic gene (transgene) only to cells of the target tissue*.* Liver-directed gene therapy has historically relied on the use of viral vectors because of their superior efficiency in delivering transgenes to cells in vivo. Due to promising characteristics associated with safety and efficacy, AAV has emerged as the most promising among viral vector candidates for preclinical gene therapy studies targeting the liver.

AAV is a single-stranded DNA virus that has been configured for use in gene therapy by removal of the endogenous viral coding sequences, allowing transgenes up to 4.7 kb to be inserted. AAV has a favorable safety profile for clinical translation as a gene therapy vector. AAVs have an absolute requirement for auxiliary genes from a co-infecting adenovirus or herpes virus for replication and are therefore helper-dependent. Additionally, wild-type AAV infections are poor activators of the immune system [8] and are not recognized to cause human disease. Because AAV is largely maintained as an episome, the risk of insertional mutagenesis is greatly reduced compared to integrating vectors, such as those based on retroviruses. AAV infection in human cells in the absence of a co-infecting virus leads to a low level of preferential integration at a locus on chromosome 19 known as AAVS1 [9]. In addition to these promising safety features, AAV can transduce both dividing and non-dividing cells, and is therefore effective in neonatal as well as adult disease models. A most powerful feature is that the AAV genome can be packaged within capsid proteins, each demonstrating distinct tropisms, which can greatly enhance the efficiency of gene transfer to specific tissues. For example, pseudotyping of the AAV2 genome with the capsid from AAV serotype 8 can endow a vector with the ability to completely transduce the liver [10, 11]. To facilitate the characterization and application of AAV, novel platforms for large-scale clinical-grade vector production have been developed [12]. While much research has been performed proving the efficacy of AAV as a gene therapy vector, more investigation is necessary to fully understand its safety profile.

Despite the great therapeutic potential of AAV vectors, several studies have shown that low levels of AAV integration into the genome do occur and can exhibit genotoxic effects [13, 14] dependent upon vector configuration and dose [14]. The outcome of murine studies on the clinical translation of AAV requires further investigation because many vector integrations that were associated with hepatocellular carcinoma (HCC) were located in a small, non-conserved microRNA (miR341). Subsequently, an unrelated study documented AAV2 integrations in known cancer genes in 11 of 193 HCCs derived from humans [15]. Because the majority of the 11 HCCs with AAV integrations derived from the livers of non-cirrhotic patients without known risk factors for HCC, the authors suggested a role of AAV2 in HCC development. Until the underlying mechanisms between AAV integrations and HCCs are fully defined, the genotoxic potential of AAV needs to be considered in future therapies relying on this vector. One solution to mitigating AAV-mediated genotoxicity would be to use genome editing to either repair the pathogenic mutation at the locus directly by targeting integration of a therapeutic cDNA into the disease gene, or correction into a “safe harbor”, a genomic locus not known to be adversely affected by insertion and expression of an engineered transgene. Thus, genome editing could mitigate the genotoxic effects of uncontrolled vector integration. New genome engineering technologies are making targeted genetic manipulations possible, and will be discussed next, with representative examples.

## Genome editing using site-specific endonucleases

**Fig. 1:**
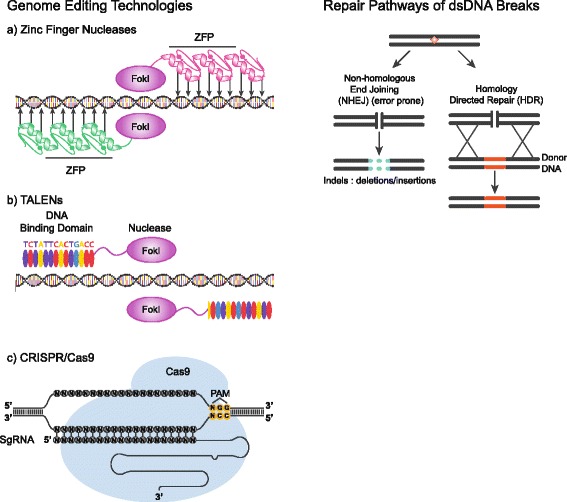
Schematic of genome engineering technologies (left) and DNA repair pathways resolving double-stranded DNA breaks (right). **a** Zinc-finger nucleases (ZFNs), **b** TALENs, and **c** the CRISPR/Cas9 system produce DNA cleavage at a desired genomic target. Once cleavage occurs, insertion of a donor template with homology to the cut site can lead to gene correction via the homology directed repair (HDR) pathway. In the absence of a donor, the random insertion or deletion of nucleotides characteristic of the non-homologous end-joining (NHEJ) pathway can result in targeted mutagenesis

The field of genome editing has evolved to address the need for improving the efficiency and specificity of traditional genome modification achieved by homologous recombination (HR). Genome engineering tools typically introduce a double-stranded break at a specific target in DNA, activating endogenous cellular repair pathways, thereby increasing the frequency of HR by several orders of magnitude [16, 17]. The double stranded break can then be repaired by non-homologous end joining (NHEJ), which leads to insertion or deletion of a small number of nucleotides (indels) at the break, or corrected via homology-directed repair (HDR), which can result in specific base-pair changes when a donor template is introduced at the site of the break [18–20]. Several genome editing technologies exist, with in vivo studies performed to date relying on zinc-finger nucleases (ZFNs), transcription activator-like effector nucleases (TALENs), or the CRISPR-Cas9 system (Fig. 1). Zinc-finger nucleases (ZFNs) were the first synthetically engineered genome editing reagents. ZFNs combine a *Fok*I restriction enzyme domain with a zinc-finger DNA binding module to target double stranded breaks in DNA [21]. *Fok*I functions as a dimer; consequently, for genome editing, ZFNs are designed in pairs that bind regions flanking the target site [22]. TALENs emerged as an alternative to ZFNs for genome editing. TALENs are similar to ZFNs and comprise a non-specific *Fok*I nuclease domain fused to a customizable DNA-binding domain. This DNA-binding domain is composed of highly conserved repeats derived from transcription activator-like effectors, which are proteins secreted by *Xanthomonas spp.* bacteria to alter gene transcription in host plant cells [23]. More recently, the clustered regularly interspaced short palindromic repeats or CRISPR-Cas9 system was identified as an RNA-guided immune system used by bacteria to protect against invading foreign viruses and other pathogens [24]. The CRISPR system from *Streptococcus pyogenes*, repurposed for genome engineering, consists of two basic components, the Cas9 endonuclease (SpCas9) and a guide RNA (gRNA). The gRNA contains a programmable recognition domain capable of binding and cleaving any genomic target proximal to a motif called the protospacer adjacent motif (PAM), which is specific to the bacterial strain from which the CRISPR system is derived. CRISPR has evolved in a few short years to become a common genome editing tool, used to manipulate genes in vitro [25–27] and even to correct disease-causing mutations in mouse models such as IEM, discussed below.

Many IEM are ideal candidates for genome editing correction because they are typically severe, liver-dominant in terms of symptoms and pathophysiology, well-defined in terms of clinical phenotypes, and yet have largely insufficient or inadequate therapy. For some, it is known that a low level of gene correction could significantly ameliorate the disease phenotype. Preclinical models of such disorders include hemophilia B [28], hereditary tyrosinemia type I [29], and ornithine transcarbamylase deficiency [30]. Hemophilia B, in particular, has been a pillar of the gene therapy field because the factor IX (*FIX*) gene fits readily into an AAV, and a low-level of protein correction leads to measurable improvements in biomarkers and restoration of hemostasis. It logically has followed that hemophilia B was the first disorder for which a preclinical mouse model was corrected by genome editing [31]. This study and subsequent studies utilizing genome editing for correction of hemophilia B as well as other IEMs are reviewed in the following section.

## Preclinical models corrected via in vivo genome editing

### Hemophilia B

Individuals most severely affected by hemophilia B exhibit circulating levels of blood clotting FIX below 1% of normal, leading to sporadic hemorrhaging from the time of birth [32, 33]. Restoration of FIX to more than 1% of normal, however, converts the disease to a more mild form, making hemophilia a powerful model to assay correction using genome editing approaches. A foundational study for in vivo genome editing used zinc-finger nucleases (ZFNs) to target the first intron of the *FIX* gene in neonatal mice, with the goal of inducing sufficient levels of HDR into the *FIX* locus [31]. A humanized murine model of hemophilia B was generated by expressing a mutant human FIX (hFIX) knocked-into the Rosa26 locus on the background of a homozygous *FIX* deletion mouse. ZFNs were delivered by a single AAV serotype 8 vector designed to target the liver, the major site of synthesis for FIX. A second AAV8 delivered a promoterless cDNA encoding exons 2–8 preceded by a splice acceptor site; this rescue cassette was engineered to recombine into the first intron of the *FIX* gene after ZFN cleavage. Upon perfect recombination, splicing of the *FIX* minigene behind the endogenous *FIX* exon 1 led to expression of functionally active FIX. In mice treated as neonates, HDR was measured at 1–3%, and FIX protein expression measured at 3–7%. Although indels produced by the ZFNs in the intron did not interfere with the creation of functional protein after HDR, the frequency of off-target cleavage by the ZFN pair, measured at up to 4%, and the lower overall efficiency of correction required that this approach be further optimized for clinical translation. Nevertheless, it was the first study to prove that genome editing could be used in vivo to correct a mammalian model of a disease.

Two studies sought to improve upon the design by directing the therapeutic transgene into albumin, an ideal safe-harbor locus due to its high transcriptional activity, and corresponding increased efficiency of integration by homologous recombination. Furthermore, a transgene correctly inserted into this locus would be expressed from the endogenous and liver-specific *Alb* promoter. Both studies aimed to introduce promoterless, partial cDNAs of *hFIX* into the albumin locus in mouse models of hemophilia B using the liver tropic AAV serotype 8 as the delivery vector, but different regions of the *Alb* gene were targeted, and different editing strategies employed.

In an extension of the genome editing study that aimed to correct hemophilia B at the disease locus, the first intron of the albumin gene was selected for insertion of a partial h*FIX* cDNA [34]. The ZFN pair targeting intron 1 was delivered on a single vector, with a second vector carrying the *hFIX* cDNA rescue cassette of exons 2 to 8. Adult C57Bl6 mice treated with 1 × 10^11^vg AAV8-ZFNs or 5 × 10^11^vg AAV8-h*FIX*-donor exhibited high circulating hFIX levels despite a low level of integration; the m*Alb*-h*FIX* mRNA corresponded to 0.5% of total wild-type m*Alb* mRNA transcripts. Although HDR commonly occurs during the S and G2 phases of the cell cycle, when cells are dividing, these results suggest that insertion of a donor sequence via NHEJ and ligation is an effective correction method in the adult liver, when cells are quiescent and more prone to DNA repair by NHEJ. Additionally, only a small number of hepatocytes need to be modified at the albumin locus in order to achieve clinically relevant levels of hFIX.

In the second study, the disruption to albumin expression was minimized by mediating HR of a corrected partial cDNA donor flanked by arms of homology without the use of site-specific endonucleases [35]. Additionally, the h*FIX* cDNA was expressed as a 2A-fusion at the end of the *Alb* reading frame, 5’ to the *Alb* stop codon. Thus, both albumin and FIX were co-expressed from a single mRNA transcript and processed into two separate proteins, both containing a signal peptide for secretion. Because neither the *FIX* nor the 2A peptide contained a methionine residue, off-target integration of the construct did not lead to hFIX expression. Targeted *Alb* alleles versus wild-type *Alb* alleles were measured at 0.5% on average for mice injected as neonates or adults at the highest dose and both neonatal and adult mice treated the rAAV8-donor showed hFIX protein levels in plasma at 7–20% of normal, with clotting activity similar to wild-type mice 2 weeks post-treatment. Targeted integration without the use of site-specific endonucleases may therefore mitigate off-target effects and the possibility of genotoxicity, but the accompanying increase in vector dose required (by up to an order of magnitude in adult mice) may pose concerns related to immune activation and manufacturing. Whether mice treated as neonates develop HCC by any of the aforementioned vectors remains to be determined, but will be critical to examine. Taken as a whole, the genome editing studies using hemophilia models demonstrate the potential for genome editing to be applied to other IEM for which a low level of corrected gene expression can lead to amelioration of the disease phenotype, such as lysosomal storage disorders.

### Hereditary tyrosinemia type I (HT-I)

HT-I is caused by mutation of fumarylacetoacetate hydrolase (FAH), the last enzyme in the tyrosine catabolic pathway. FAH deficiency causes accumulation of fumarylacetoacetate in hepatocytes and results in severe liver damage. Affected individuals fail to gain weight and grow at the expected rate, which is recapitulated by the *Fah* ^–/–^ mouse model [29, 36]. Current therapies for HT-I include nitisinone (NTBC), which inhibits hydroxyphenylpyruvate dioxygenase (HPD), the enzyme catalyzing the second step of tyrosine catabolism. Removal of NTBC from the diet of mutant mice results in the expansion of repaired hepatocytes that repopulate the liver, a unique characteristic of this disease model which makes it useful for testing the efficacy of novel genome editing therapies [36], because it allows selection of cells that have been corrected in vivo. In the first study using CRISPR to correct a disease model in vivo [37], CRISPR components were systemically delivered via hydrodynamic tail vein injection in a mouse model of hereditary tyrosinemia type I. The Cas9 enzyme from *S. pyogenes* and one of three gRNAs (FAH1, FAH2 or FAH3) were co-injected with a short single-stranded DNA oligo donor template into adult *Fah* ^*–/–*^ mice. Animals receiving the FAH2 guide targeting in the intron just downstream of the point mutation did not lose weight after NTBC-containing water was withdrawn, while untreated *Fah* ^*–/–*^ mice experienced rapid weight loss after NTBC withdrawal, and then death. Due to the selective advantage conferred after successful editing at the *Fah* locus, a correction of 0.4% was sufficient to rescue the treated mutant mice from lethality; 30 days after NTBC withdrawal, 33.5% of hepatocytes were Fah^+^ in treated mice. Follow-up studies aimed to improve the efficacy and mode of delivery for clinical implementation. By coupling transient non-viral delivery methods with pharmacological selection, a follow-up study was able to achieve a more than ten-fold increase in corrected hepatocytes [38].

In a complementary approach using AAV gene delivery, HDR-mediated rescue of a point mutation in the *Fah* gene was accomplished using an AAV8 vector to deliver the FAH2 guide RNA and a HDR template (AAV-HDR) [38]. The *SpCas9* mRNA was packaged into a lipid nanoparticle (nano.Cas9) for short-term expression, reducing the chance of genotoxic off-target effects. Fourteen days post-treatment, a greater than 6% correction of Fah^+^ hepatocytes was detected in treated *Fah* ^*–/–*^ mice. Highly efficient delivery vectors led to a significant increase in the initial correction of Fah over the initial treatment, and completely restored the liver by 1 month.

An alternative approach has leveraged the potent gene inactivating capability of the CRISPR system to target an enzyme upstream of FAH in the tyrosine catabolic pathway, HPD, as an alternative method of correcting the *Fah* ^*–/–*^ mouse model. Metabolic reprogramming by knockout of the HPD enzyme would convert HT-I to a milder disorder, HT-III. As predicted, *Fah* ^*–/–*^ *; Hpd* ^*–/–*^ mice show reduced risk of HCC compared to *Fah* ^*–/–*^ mice treated with NTBC; thus, bypassing this pathway via deletion of the *HPD* gene could be an effective treatment for HT-I patients [39]. To test this premise, CRISPR components designed to inactivate HPD were delivered to adult *Fah* ^*–/–*^ mice [40]. Assessment of editing efficiency by staining cells for Hpd expression showed that bi-allelic knockout at 1 week post-treatment was 8%. At 4 weeks, the efficiency increased to 68% due to the positive selection of *Hpd* ^*–/–*^ hepatocytes, and treated mice phenotypically resembled wild-type mice at 25–27 days post withdrawal of NTBC. In some mice, healthy hepatocytes completely replaced diseased cells by 8 weeks post-treatment. As with direct correction at the *Fah* locus, the success of this strategy was contingent upon the positive selection inherent to the HT-I model, and provided the incentive for testing artificial methods of induced selection to enrich for desired editing events in modified hepatocytes.

Improving the efficacy of genome editing is a pervasive problem independent of targeting strategy and delivery method. Although the previous examples are encouraging, they are applicable only in the rare setting in which healthy hepatocytes have a selective growth advantage, such as in HT-I. For most liver diseases, this is not the case, and a method of selecting for cells would allow the amplification of less efficient gene correction. To this end, a novel approach using a small molecule and gene editing has been used to induce a transient state of superior fitness in corrected hepatocytes. Critical to the success of this study has been the use of a transition state analogue and potent inhibitor of FAH, CEPHOBA, which can induce a transient HT-I [41], and the previous observation that shRNA-mediated knockdown of an alternate enzyme in the pathway, Hpd, can positively select hepatocytes in the HT-I mouse model [42]. To recapitulate positive selection in wild-type mice, an rAAV8 cassette was designed with an *Hpd* shRNA (sh*Hpd*) embedded in a miRNA so that it would be expressed only after perfect recombination into the albumin gene. Co-delivery of the hFIX cDNA fused to a 2A peptide on the same vector allowed for facile assaying of HDR events at the *Alb* locus*.* The dual expression construct was administered to neonatal C57Bl6 mice, and at 4 weeks of age, CEPHOBA was injected daily for 4 weeks to enrich for gene targeted cells. In two of three mice, hFIX levels steadily increased and were stable in the therapeutic range but declined in a third. With a second round of CEPHOBA, however, hFIX was expressed in the therapeutic range for all three mice. shRNA-mediated knockdown of *Hpd* thus protected hepatocytes against the effects of the FAH inhibitor CEPHOBA. Although efficiency of HR with this method was less than 1%, after selection, transgene-expressing cells constituted 50% of the liver. Any transgene delivered in cis to the protective shRNA could be co-selected, making the “GeneRide” construct a malleable instrument applicable to other metabolic diseases, with fewer concerns of off-target integration.

### Ornithine transcarbamylase (OTC) deficiency

OTC deficiency, an X-linked disease, is the most common urea cycle disorder. Affected individuals generally possess little or no functional OTC enzyme, but restoration of as little as 3% activity can significantly improve the disease phenotype [43]. The *spf* ^*ash*^ OTC mouse model recapitulates the hyperammonemia commonly seen in patients when stressed with a high protein diet, due to residual levels of OTC activity (~5%) in these mice [30]. To accomplish therapeutic editing in vivo at the *Otc* locus, an SaCas9/gRNA system was used to correct a disease-causing mutation in the *spf* ^*ash*^ mice [44]. SaCas9 under expression of the liver-specific TBG promoter was packaged into a serotype-8 capsid and co-delivered with a second AAV8 carrying the gRNA cassette and a donor template. In mice treated as neonates, 10% of *Otc* alleles were corrected and Otc enzyme activity was restored to 20% and 16% at 3 and 8 weeks, respectively, in liver homogenates. All of the mice treated as neonates survived a 1-week high protein stress test, and manifested significantly lower ammonia levels compared to untreated *spf* ^*ash*^ mice, where one-third of control *spf* ^*ash*^ mice died or had to be euthanized when treated in an identical fashion. In comparison to the neonatal mice, *spf* ^*ash*^ mice treated as adults exhibited higher levels of NHEJ, with deletions large enough to extend into the adjacent exon. Although all treated adults displayed effects of Otc deficiency, the higher doses of AAV were more severe, culminating in termination of the experiment at 2 weeks. These animals showed elevated levels of plasma ammonia, suggesting that editing presumably led to increased mutations in the Otc gene, causing substantial loss of Otc activity and leading the mice to succumb to the resultant hyperammonemia. Such an outcome emphasizes the importance of further characterizing DNA repair in vivo after genome manipulation.

### Lysosomal storage disorders

The proof-of-concept studies reviewed above clearly demonstrate the therapeutic potential of genome editing approaches. Future success will depend on identifying and implementing correction strategies applicable to a broad range of disorders, such as insertion of a therapeutic transgene at a safe harbor locus. The same system using ZFNs to insert a cDNA into the albumin locus also led to a similar approach to treat two lysosomal storage disorders [34]. Preclinical studies performed in Hurler and Hunter syndrome mouse models demonstrated high activity levels of alpha-L-iduronidase and iduronate sulfatase, respectively, in liver and plasma post-treatment with a single dose of two AAVs – one to deliver the *Alb* ZFNs, and the other a cDNA donor to correct the enzyme defect. A unique feature of certain lysosomal storage disorders is that cross correction, i.e., the restoration of enzyme activity in uncorrected tissues that take up the secreted enzyme from the circulation, can be observed. The data suggests that therapeutic levels of alpha-L-iduronidase and iduronate sulfatase produced in the liver could have widely beneficial effects.

In addition to albumin, there is the potential for integration into other safe harbor loci. Targeting of the glucose-6-phosphatase (*G6PC*) cDNA into the *Rosa26* locus using ZFNs led to increased survival in a mouse model of glycogen storage disease type Ia (GSD1a) [45]. A dual AAV system was employed, with one vector delivering ZFNs and a second the *G6PC* transgene. While 8-month-old knockout mice showed improved survival, from 43% to 100%, when components were delivered with serotype 8 vectors, correction of standard GSD1a biomarkers was observed only when components were delivered via AAV serotype 9. Interestingly, there appeared to be a selective advantage for stably transduced G6P-ase expressing hepatocytes, as determined by an increase in allele modification for treated knockout mice versus treated wild-type littermates, a phenomenon not previously observed. This study establishes *Rosa26* as a safe harbor locus for transgenesis via gene targeting in alternate murine disease models. In human-derived models, parallel efforts have successfully integrated therapeutic cDNA into the AAVS1 site in stem cells [46] and T cells [47]; the ongoing transcriptional activity of transgenes inserted in AAVS1 makes it a plausible target [48]. Murine and rodent models have been generated carrying the AAVS1 locus [49] and could serve to test future AAVS1-targeting genome editing platforms. Table 1 summarizes the studies performed to date that have successfully employed in vivo genome editing to correct or ameliorate disease manifestations in mouse models of IEMs.

**Table 1:** Summaries of genome editing studies performed on preclinical models of inborn errors of metabolism

IEM	Summary	Comment	Reference
Hemophilia B	Targeting *FIX* disease locus Dual AAV8 vectors: Zinc-finger nucleases (ZFNs) Partial FIX cDNA Neonatal, hemophilia B mouse model	First in vivo study using therapeutic genome editing Low rates of homology-directed repair (HDR) detectable off-target events (<4%)	Li et al., 2011 [33]
Hemophilia B	Targeting *Alb* safe harbor Dual AAV8 vectors: ZFNs Partial FIX cDNA Adult C57BL/6+ hemophilia B mouse models	Low rate of NHEJ-mediated correction (0.5% fused mRNA transcripts) Due to integration in *Alb*, secretion of corrected protein led to therapeutic levels of circulating FIX Duration of effect 12 weeks after single treatment	Sharma et al., 2015 [34]
Hemophilia B	Targeting *Alb* safe harbor No endonuclease Single AAV8 vector targeting FIX donor as 2A fusion to *Alb* Neonatal + adult hemophilia B mice	No off-target Low rate of HDR (0.1% fused mRNA transcripts) Addition of amino-terminal proline to FIX due to 2A peptide	Barzel et al., 2015 [35]
Hereditary Tyrosinemia Type I	Targeting *Fah* disease locus CRISPR: SpCas9/gRNA + ssDNA oligonucleotide Naked DNA	Positive selection of hereditary tyrosinemia type I (HT-I) mouse model Low HDR (0.4%); increased to 33.5% after 30 days Off-target for *Fah* gRNA < 0.3% (NIH3T3 cells)	Yin et al., 2014 [81]
Hereditary Tyrosinemia Type I	Targeting *Fah* disease locus CRISPR: SpCas9 mRNA, LNP delivery (nano.Cas9) FAH2 gRNA + donor (AAV-HDR)	Transient expression SpCas9 (LNP) HDR 6% without selection Low level off-target cutting (Hepa1-6 cells)	Yin et al., 2016 [37]
Hereditary Tyrosinemia Type I	Targeting *Hpd* (disease locus) CRISPR: SpCas9 + 2 gRNAs non-homologous end joining (NHEJ) Naked DNA	Positive selection of HT-I mouse model Metabolic reprogramming Multiplex editing (2 guides) 8% NHEJ efficiency at both cut sites 1-week post 68% efficiency 4-weeks post (+ selection)	Pankowicz et al., 2016 [39]
Hereditary Tyrosinemia Type I	Targeting *Alb* safe harbor No endonuclease rAAV8: *Hpd* shRNA + hFIX C57Bl/6 wild-type mice	Inducible positive selection using CEPHOBA Initial homologous recombination < 1%; after selection 50%	Nygaard et al., 2016 [41]
Ornithine Transcarbamylase Deficiency	Targeting *Otc* disease locus CRISPR: SaCas9 + gRNA Dual AAV8 vectors: SaCas9 + gRNA-donor *Otc* mouse model	Smaller Cas9 orthologue *S. aureus* 10% HDR in neonatal mice Large deletions in adult mice ➔ hyperammonemia	Yang et al., 2016 [43]
Lysosomal Storage Disorders (MPSI, MPSII)	Targeting *Alb* safe harbor Dual AAV8 vectors: ZFNs + donor C57Bl/6 wild-type mice	Therapeutic protein detectable by Western blot Phase I clinical trial MPSI: 3 AAV6 vectors: ZFN + ZFN + donor	Sharma et al., 2015 [34]
Glycogen Storage Disorder Type Ia	Targeting *Rosa26* safe harbor Dual AAV8/AAV9 ZFNs + donor GSD1a mouse model	AAV8: Improvement in survival AAV9: Improvement in biochemical phenotype Positive selection of corrected hepatocytes	Landau et al., 2016 [44]

## Alternative strategies – the genome editing toolbox

### Improvements to CRISPR and new reagents

Expression of genome editing components in a manner that is both efficient and non-toxic to the cell presents a barrier to application of most editing systems in vivo. The well-characterized CRISPR/Cas9 from *S. pyogenes* is encoded by a cDNA of 4.2 kb, and while feasible to package in an AAV, is too large for inclusion of the regulatory elements that would lead to enhanced levels of protein expression. By identifying and characterizing orthologous Cas9 proteins such as those from *S. thermophilu*s (StCas9) [50], *N. meningitidis* (NmCas9) [51], and *S. aureus* (SaCas9) [52], which are smaller in size but show similar activity to the SpCas9, the *Cas9* nuclease can now be packaged efficiently.

The identification and characterization of distinct CRISPR systems could expand the genome editing toolbox to engineer sites not previously amenable. The well-studied Cas9 CRISPR systems are classified as class 2 type II systems. A putative class 2 type V system has been identified, possessing an endonuclease domain known as Cpf1 [53]. Unlike Cas9, Cpf1 introduces a staggered, double-stranded DNA break distal from the PAM. Insertion of a donor template having complementary overhangs to such a staggered break could be significantly more efficient than repair of the blunt-ended DNA breaks produced by Cas9, leading to increased hepatocyte correction in disease models. Two Cpf1 orthologs were initially characterized and shown to have activity in a human HEK293 cell line. These orthologs have been further assayed in two independent studies for their on-target activity and genome-wide specificity [54, 55]. Both studies showed that the Cpf1 endonuclease from *Lachnospiraceae bacterium* (LbCpf1) and *Acidaminococcus sp* (AsCpf1) exhibit robust mutagenicity in human cell lines. Sites in the *DNMT1* gene were assayed for on-target specificity. Both studies identified nucleotides 1–18 downstream from the PAM as necessary for Cpf1 activity; double mismatches in this region abrogated Cpf1-mediated indels. Both studies also found that, at the sites assayed, Cpf1 showed greater fidelity than SpCas9 without being less efficient. Whether AsCpf1 and LbCpf1 are as effective at cleaving DNA targets at other sites in the genome, and with superior specificity, remains to be investigated.

In addition to DNA editing, new native and synthetic CRISPR systems are being identified and engineered for manipulating RNA. *L. shahii* C2c2 is a putative class 2 type VI system which cuts RNA via two HEPN RNase domains [56]. C2c2 cleaves ssRNA dependent on target sequence and secondary structure. Interestingly, C2c2 is activated not only to cleave its intended target, but once active, indiscriminately cleaves RNA in a non-specific manner culminating in programmed cell death (PCD). A more detailed understanding of the mode by which C2c2 induces PCD would allow C2c2 to be used to trigger PCD or dormancy in specific cells such as cancer cells expressing a particular transcript or cells infected by a specific pathogen. The application of C2c2 in this manner would require absolute on-target specificity.

Elucidation of the mechanisms by which Cas9 targets and binds DNA has led to engineering of novel Cas9 variants with improved range or improved specificity. By manipulating the PAM recognition domain, Cas9 variants with expanded target ranges have been generated [57, 58]. Additionally, amino acid mutations have been introduced in the endonuclease to reduce non-specific DNA binding, improving specificity [59]. Capitalizing on CRISPR’s robust DNA-recognition ability, a new technology executes conversion of a single DNA base pair from cytosine to uracil [60]. This technology, designated as base editor, fuses the cytidine deaminase enzyme APOBEC1 to a catalytically inactive Cas9 (dCas9) endonuclease (BE1). Using insights from CRISPR’s DNA binding and cleavage mechanisms, BE1 has been enhanced in subsequent generations (BE3). The BE3 system successfully corrected a single base pair mutation after nucleofection in two cell culture disease models, at higher efficiencies and with lower indel formation than the wild-type SpCas9 plus a donor ssDNA. A hindrance to the application of this technology is that the base editor converts any cytosine in the target site, thereby requiring strict target selection. Additionally, off-target indels are replaced by off-target C-U conversions that might be more frequent given the higher efficiency compared to SpCas9 at the on-target site. Further adjustment to reduce the base pair conversion window, combined with careful target selection, would be required to refine this technology for translational use.

### Tissue-specific genome editing

Genome editing in the brain and other tissues of the central nervous system is of paramount interest for neurodegenerative diseases as well as lysosomal storage disorders, but gene transfer has proven difficult due, in part, to the blood brain barrier. An in vivo study quantified the efficiency and nature of indels in primary neurons after AAV-mediated delivery of SpCas9 [61]. MeCP2, a protein ubiquitously expressed in the brain and implicated in Rett syndrome, was successfully knocked-down in adult male C57BL6 mice, with indel levels as high as 67.5%. Multiplex genome editing from a dual-AAV vector system enabled concurrent knockdown of *Dnmt1*, *Dnmt3a*, and *Dnmt3b*, genes responsible for memory formation and learning*.* Simultaneous editing of the three loci was observed in approximately 35% of all transduced neurons with only 0–1.6% off-target cleavage. The ability to manipulate multiple genes in a tissue-specific context, and in the brain in particular, should allow for further elucidation of disease-related gene function and could ultimately lead to simultaneous correction of multiple mutations.

Duchenne muscular dystrophy (DMD) is a progressive muscle degenerative disease caused by mutations in the dystrophin gene that disrupt the reading frame and lead to loss of functional dystrophin expression. Milder forms of DMD are caused by in-frame deletions in the dystrophin gene, resulting in expression of a truncated but partially functional protein. Therapies that aim to restore the dystrophin reading frame could therefore be effective. Three independent studies used CRISPR to restore the reading frame in the *mdx* mouse model of DMD, which expresses a truncated dystrophin [62]. In one study, two AAV8 vectors separately delivered *SaCas9* and two gRNAs targeting the intronic regions flanking the mutated exon 23. Exon 23 was shown to be deleted from 2% of alleles, with only 3% indels at the target site and less than 1% indels at the top off-target site for both guides [63]. A second study used a similar design but delivered CRISPR components using a serotype 9 vector, which efficiently transduces skeletal and cardiac muscle. SaCas9 activity was enhanced by modification of the gRNA scaffold, which led to improved exon 23 excision (39% by deep sequencing) with minimal off-target activity [64]. A third study used AAV9 vectors, but delivered the SpCas9 under expression of a truncated promoter, with the two guide RNAs on a separate vector. Dystrophin expression at up to 25% was restored in myofibers as measured by immunostaining [65]. Dystrophin expression as low as 3–15% can alleviate pathogenesis in cardiac and skeletal muscle; thus, these studies demonstrate the benefit of using CRISPR for in vivo multiplex editing of disease models showing tissue-specific pathology.

## Conclusions

### Hurdles and future directions for genome editing therapeutics: the 0.1%

As CRISPR-Cas9 approaches the clinic, understanding the host response to the Cas9 protein and AAV vector components is essential. Delineating these responses and developing strategies to manage them will be critical to achieving clinical success. Although AAV are capable of priming CD8+ T cell responses towards a transgene, lack of co-stimulatory signals can result in impaired proliferation and cytokine secretion, generating passive tolerance [66–68]. However, a certain degree of inflammation due to factors such as serotype can yield a robust CD8+ response [69]. A recent study attempted to characterize the host response to AAV9-delivery of a split Cas9 system, for which the Cas9 coding sequence was distributed between two vectors and reconstituted post-translationally [70]. Cas9 expression in the tibialis interior muscle of adult male C57Bl6 mice increased the frequency of antigen-specific T-cells among injected mice; Cas9-specific antibodies were also observed. The vector stimulated AAV capsid-specific antibodies, but in contrast to Cas9, the epitopes were shared between individually treated animals. Two weeks after administration, no significant muscle cell damage or repair response was observed despite the presence of CD8+ T cells. Managing the host response to AAV-delivery of CRISPR components will rely on understanding the implications of immune activation when cell damage may yet be undetectable. Determining whether host immune response to AAV-Cas9-CRISPR interferes with on- and off-targeting, as well as studying the host response in clinically relevant disease models, requires further study.

An important safety issue for genome editing is the accurate assessment of off-target cleavage by endonucleases and mitigating the effects of non-specific activity. Understanding the interaction between synthetic editing systems and DNA cleavage has led to the development of algorithms to appraise likely off-target activity [71–73]. In general, genome editing systems found to be highly effective at mediating on-target cleavage conversely tend to show increased off-target activity. High fidelity Cas9 variants have reduced on-target activity and non-specific interaction with the DNA backbone thereby diminishing the overall affinity of a Cas9/gRNA complex for a particular on-target or off-target site [59]. This is important for gene repair systems relying on HDR, where a high number of cutting events must occur to achieve even a modest level of therapeutic correction. In a recent study targeting correction of the β-globin gene implicated in sickle-cell disease, hematopoietic stem/progenitor cells were edited ex vivo using a ribonucleoprotein complex of recombinant Cas9 protein and an in vitro transcribed gRNA [74]. Despite appreciable levels of HDR in CD34^+^ hematopoietic stem/progenitor cells, off-target activity of up to 80% was measured at the top-scoring site (OT1), with chromosomal translocations occurring between the on-target and off-target sites. To reduce off-target effects, two high-fidelity Cas9 variants were tested. These variants did not lead to indel formation at OT1, but exhibited a five-fold decrease in indels at the on-target site, leading to the use of the wild-type Cas9 for further experimental use. Another approach to reducing or eliminating off-target activity is through the use of the StCas9, NmCas9, and SaCas9 orthologs. These orthologs recognize longer PAM sequences relative to the widely used SpCas9 system, and therefore have a longer recognition sequence. This increased length requirement reduces the frequency of potential off-target sites within the genome and several studies have shown that these Cas9 orthologs have less off-target effects when tested in a direct comparison with SpCas9 [75–77]. Until more powerful high-fidelity Cas9 variants are engineered, the use of promiscuous wild-type Cas9 endonucleases in combination with assaying for off-target cleavage remains the method of choice for gene correction studies.

Although adeno-associated viral vectors have been utilized substantially for in vivo delivery of genome editing components, limitations are associated with these vectors, including reduced DNA packaging capacity and the potential for long-term expression of the dispatched endonuclease. Non-viral gene therapy vectors have the potential to address these limitations, particularly with respect to safety. Vectors such as lipid nanoparticles formulated with improved polymers can lead to transient in vivo expression of the well-characterized *SpCas9*, reducing the hazards posed by off-target effects and host immune response while improving genome editing efficiency [38]. Alternative delivery of CRISPR components as ribonucleoproteins with single-stranded DNA donors would be a more economical method of rapid, highly efficient gene manipulation [74]. Enhancement and innovation of non-viral delivery of genome editing reagents could address many of the current safety issues surrounding the in vivo applications of CRISPR-Cas9 technology.

Correction of a disease-causing mutation from a single treatment given at birth is the goal towards which genome editing therapies for IEM are directed. The widespread implementation of newborn screening has made it possible to detect a number of IEM at a pre-symptomatic stage when medical intervention has the ability to alter the natural history of the disease. Now, as the field of genome editing matures, the objective of the field appears within reach; specifically, the correction of a mutation at the endogenous locus. To that end, the rapid development of highly specific Cas9 orthologs that can be efficiently packaged into AAV is encouraging as are improvements in non-viral delivery platforms. However, current genome editing practices have shortcomings – most rely on cellular repair pathways that are not well understood, leading to low efficiency of correction and an unintended alteration of non-targeted coding sequences. A more thorough comprehension of DNA repair mechanisms after cleavage by Cas9 and other CRISPR systems should improve genome editing specificity, while the development of novel selection strategies that give a competitive advantage to gene-corrected cells could reduce some of the issues with efficiency.

Already, a better understanding of CRISPR mechanisms has led to improvements in the technology. Temporal control over CRISPR expression has been achieved with the development of a chemically inducible CRISPR system [78]. By modifying Cas9 to high-fidelity variants, as well as fusing catalytically inactive Cas9 to a *Fok*I endonuclease domain, the specificity has improved [59, 79]. In a Cas9 devoid approach, triplex-forming peptide nucleic acids have mediated HDR repair, and when delivered via polymer nanoparticles with a donor template, enabled up to 4% gene editing in thalassemic mice [80]. Recently, genome editing has been applied in the clinic to HIV [80], a fatal infection whose current treatment regimen involves a cocktail of toxic drugs, taken for the life of the patient. ZFNs designed to eradicate expression of chemokine receptor 5 (CCR5), a co-receptor commonly used by HIV for entry into CD4 T cells, reportedly increased the median CD4 T cell count by three-fold in study participants, augmenting their natural anti-viral immunity [81]. Initial therapeutic applications of genome editing such as this one have relied on its effectiveness at mediating gene knockout; achieving precise gene correction will require improvements. As CRISPR technology upgrades, the enhancement to efficiency and safety will bring genome editing closer to the clinic for patients with IEMs, especially those in need of improved therapies.
